# An in-vivo experimental evaluation of the efficacy of fish-derived antimicrobial peptides against multidrug-resistant *Pseudomonas aeruginosa*

**DOI:** 10.11604/pamj.2023.46.112.38578

**Published:** 2023-12-21

**Authors:** Agharid Ali Al-Rasheed, Bashiru Garba, Kareem Obayes Handool, Karim Alwan Al-Jashamy, Mohamed Naji Ahmed Odhah, Najib Isse Dirie, Hassan Mohd Daud

**Affiliations:** 1 Department of Microbiology, Faculty of Veterinary Medicine, Tikrit University, Tikrit, Iraq,; 2 Department of Clinical Studies, Faculty of Veterinary Medicine, Universiti Putra Malaysia, Serdang, Selangor Darul Ehsan, Malaysia,; 3 Department of Veterinary Public Health and Preventive Medicine, Faculty of Veterinary Medicine, Usmanu Danfodiyo University, Sokoto, Nigeria,; 4 Department of Veterinary Directorate, Ministry of Agriculture, Baghdad, Iraq,; 5 Department of Radiology and Sonar Technology, Bilad Alrafidain University College, Baghdad, Iraq,; 6 Department of Veterinary Public Health, Faculty of Veterinary Medicine, Thamar University, Dhamar, Yemen,; 7 Department of Urology, Dr. Sumait Hospital, Faculty of Medicine and Health Sciences, SIMAD University, Mogadishu, Somalia

**Keywords:** Antimicrobial peptides, *Pseudomonas aeruginosa*, antimicrobial resistance, serum biochemistry, histopathological analysis

## Abstract

**Introduction:**

due to the fact that antimicrobial peptides antimicrobial peptides (AMPs) from climbing perch have not been fully explored for their antimicrobial potency, this investigation was undertaken to explore that possibility.

**Methods:**

antimicrobial peptides (AMPs) from the mucous secretion of climbing perch were obtained and an in-vivo analysis was conducted using mice.

**Results:**

the results showed inhibitory effects on multidrug-resistant multidrug-resistant Pseudomonas aeruginosa with reduced mortality from 100% among the non-treated group to 25%. Similarly, the level of serum transaminase enzymes (AST and ALT), creatinine levels, and pro-inflammatory cytokines (TNF-α and IL-6) were all found to be higher in the non-treatment group compared to the AMP-treatment group. Also, extensive tissue damage in the lung, liver, and spleen of the non-treated control group mice was observed based on the histopathological lesions recorded. As expected, AMPs from climbing perch significantly alleviated multidrug-resistant P. aeruginosa infection in-vivo and produced enhanced therapeutic efficacy superior to the ciprofloxacin treatment.

**Conclusion:**

this study provides insight into the potential antimicrobial activity of fish innate immune system-derived peptides that could serve as a candidate for the substitute of antibiotics.

## Introduction

The popularity of naturally occurring antimicrobial compounds is fast increasing globally. The attention that these compounds are getting is because of the dramatic rise of microorganisms including foodborne pathogens that have become resistant to synthetic antimicrobials used for their treatment [1]. A number of these natural compounds with valuable and clinical antimicrobial activity are obtained from medicinal plants, and marine and terrestrial organisms, such as fungi and bacteria [2,3]. Many studies have been conducted to evaluate the potential of these natural antimicrobial compounds as an alternative to synthetic commercial drugs due to the emergence of multidrug-resistant MDR strains of the pathogens. These include freshwater and marine organisms which are rich sources of biologically active metabolites [4]. Antimicrobial peptides (AMPs) are a class of small peptides occurring in nature and are an important component of the innate immune system of many organisms. Considerable progress has been recorded with vertebrate AMPs for usage in topical applications and a few AMPs have been utilized in clinical trials [5-7]. However, few works have been done to study the efficacy of fish antimicrobial peptides against *P. aeruginosa*. Antimicrobial proteins and peptides from the mucus of climbing perch could be used as natural antimicrobial agents for internal usage.

Pseudomonads are a group of bacterial pathogens causing life-threatening ulcerative syndrome, abdominal infections, cystic fibrosis, and hemorrhagic septicemia [8-10]. Although *Pseudomonas aeruginosa* is a part of normal fish microbiota, under stressful conditions (such as poor water quality, temperature, diet composition, malnutrition, and overcrowding) the bacteria can become pathogenic, causing serious illnesses [11]. The management of these diseases is mostly achieved by the application of antimicrobial agents. However, in recent years, the extensive use of antimicrobial agents in animal husbandry including the aquaculture sector has led to the development and emergence of multidrug-resistant bacterial pathogens which constitute a serious public health challenge globally [12-15]. Despite the undesirable effects associated with the use of antimicrobials in the treatment of fish disease, antibiotics are still the most favorable strategy to control bacterial infection in fish [16].

This study aims to evaluate the efficacy of antimicrobial proteins and their potential bioactive peptides obtained from climbing perch against multi-drug resistant *P. aeruginosa* using mice.

## Methods

**Study design:** an experimental study approach was used where a total of forty-four male ICR mice weighing 21.2 ± 0.75 g were purchased from the Animal Resource Unit, Faculty of Veterinary Medicine, and Universiti Putra, Malaysia. The mice were housed at the animal research facility at the Faculty of Veterinary Medicine fed with standard commercial pelleted feed and water *ad-lib* with potable water and were arbitrarily assigned to groups after seven days of acclimatization.

**In-vivo virulence evaluation of *P. aeruginosa*:** virulence was determined by infecting mice with *P. aeruginosa* according to Reed and Muench (1938) [17]. Four groups with five mice each were challenged with different doses of *P. aeruginosa* (CFU/mL) equivalent to 0.5, 2.0, 4.0 and 8.0 McFarland standards (1x108, as well as 5x107, 2.5x107, and (1.25x107). The inoculum size was 0.2 mL and the mice were injected intraperitoneally using a 1 mL 27G syringe. The clinical sign of illness and mortality were recorded through 48 h. The percentage of mortality rates was calculated by the total number of death and surviving mice for each dose as reported by Reed and Munch *et al*. (1938). LD50 was calculated from the log number of *P. aeruginosa* for each dose against the mortality rates of mice.

**Preparation and purification of peptide:** the antimicrobial peptide (AMP) was prepared from healthy climbing perch purchased from a local wet market in Seri Kembangan, Malaysia. The fish (15) were initially kept in an aerated aquarium to acclimatize for one week before they were subjected to hypothermic stress as reported by Agharid *et al*. [6]. The mucus excreted was collected and lyophilized at -80°C under vacuum at 0.018 pressure in a vacuum freeze-dryer, before 200 mg of the lyophilized epidermal mucus was dissolved in a 1% acetic acid in 1: 4 ratios and then heated in a boiling water bath to inhibit proteolytic enzymes and solubilize the low molecular weight peptides [18]. Subsequently, the acetic acid mixture was heated mixture was completely homogenized (Polytron® Homogenizer) on dry ice for 5 min. The homogenate was then centrifuged at 4°C for 35 minutes at a speed of 15,000 rpm. Finally, the supernatant (antimicrobial proteins and bioactive proteins in crude (AMPPC)) was aspirated into a 0.45 μm Whatman No.1 filter paper and then stored at 4°C until required.

**Figure 1:**
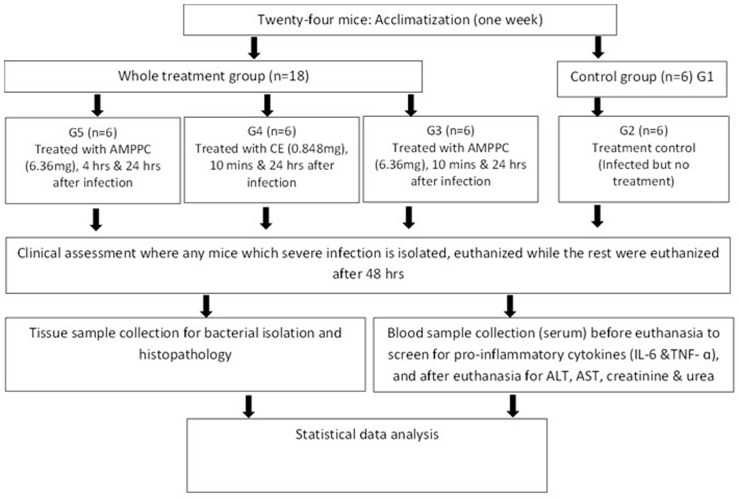
flowchart showing the experimental animal procedure

**Efficacy of peptides against *P. aeruginosa* sepsis:** twenty-four Institute of Cancer Research (ICR) mice (21-22 g) randomly placed in four groups of six each were orally administered with 200 μl containing 2.5 × 107 CFU/ml of multi-drug resistant *P. aeruginosa*. After the bacterial challenge, the mice in each group were treated with phosphate-buffered saline (PBS) (group G2); 6.36 mg/mouse of AMP (group G3) twice (after 10 min and after 24 h of infection); one dose of ciprofloxacin (CE) (0.848 mg/mouse) for group G4 10 min after infection; and group G5 was treated with AMP (6.36 mg/mouse) 4 h before and 1 day after infection. Food was withdrawn for 3-4 hours from the mouse before treatments (Figure 1).

The mice were monitored at least four times daily and the rate and status of survival were recorded for every 6 h until 48 h. The assessment of treatment by AMP and CE was measured and compared with the PBS control group through the bacteria isolation, biochemical parameters (liver enzymes and renal function), histopathological investigation, and evaluation of immune response.

**Isolation and identification of *P. aeruginosa*:** a loop-full sample of blood and homogenate from the collected internal organs (liver, lung and spleen) was directly streaked onto MacConkey agar (MCA) (Oxoid CM0007, Thermo Fisher Scientific) in duplicate and incubated at 37°C for 24-48 h under aerobic condition. Colonies were counted and expressed as CFU/g for organs and CFU/mL for blood. The production of yellowish-green, fluorescent pigment is commonly associated with pseudomonads [19].

**Serum biochemical analysis:** blood samples collected via exsanguination under general anesthesia were analyzed for concentrations of aspartate transaminase (AST), alanine transaminase (ALT), urea, and creatinine using an automatic chemistry analyzer machine (Hitachi 902 Automated Analyzer).

**Immunological assays:** the concentrations of pro-inflammatory cytokines; Tumor Necrosis Factor (TNF-α) and Interleukin-6 (IL-6) were determined using a commercially available kit (Elabscience Biotechnology, USA, Catalog No: E-EL-M0044) according to the manufacturer´s protocol. One hundred microliters of mouse serum samples and IL-6 standards in serial concentrations of 1000, 500, 250, 125, 62.5, 31.2, 15.6, and 0 pg/mL were added in duplicate to each well coated with IL-6-specific antibody and incubated for 90 min at 37°C. Then, the serum was removed, and 100 μL of biotin labelled with IL-6-specific antibody conjugate was added and incubated for 1 h at 37°C. Each well was aspirated and washed three times with washing buffer. Horseradish Peroxidase (HRP)-avidin (100μL) was added to every well and incubated for 30 min at 37°C and the plate-washing process was repeated five times using wash buffer. The plate was blotted on a clean filter paper for absolute removal of washing fluid and the tetramethylbenzidine (TMB) substrate (90μL) was added to every well and incubated for 15 min at 37°C. Stop solution (50μL) was added to every well and the ELISA reader was set at 450 nm to immediately determine the optical density. A standard curve was prepared from seven IL-6 standard dilutions and IL-6 concentration in serum samples was determined. Similarly, tumor necrosis factor-α (TNF-α) concentration was evaluated through commercially available Enzyme-Linked Immunosorbent Assay (ELISA) kit (Elabscience Biotechnology, USA, Catalog No: E-EL-M0713) following the manufacturer´s guideline. One hundred microliters of mouse serum and TNF-α standards in serial concentrations of 1000, 500, 250, 125, 62.5, and 31.20 pg/mL were added in duplicate to every well coated with TNF-α-specific antibody and incubated for 90 min at 37°C. Then, the serum was removed and 100 μL of biotin labelled with TNF-α-specific antibody conjugate was added. The plate was incubated for 1 h at 37°C. Every well was aspirated and washed three times with washing buffer. HRP-avidin (100 μL) was added to every well and incubated for 30 min at 37°C. The plate was washed, and the procedure was repeated five times with washing buffer. Next, the plate was blotted on a clean filter paper for complete removal of wash fluid, TMB substrate (90 μL) was added to each well, and the plate was incubated for 15 min at 37 °C. Stop solution (50 μL) was added to each well, and the optical density was determined immediately with an ELISA reader set at 450 nm. A standard curve was prepared from seven TNF-α standard dilutions and TNF-α concentration in serum samples was determined. The detection limits for IL-6 and TNF-α were 31.25-2000 pg/mL and 7.81-500 pg/mL respectively.

**Histopathology:** the internal organs (liver, spleen, and lung) were harvested, fixed, and preserved in 10% buffered formalin solution (Fisher Scientific) for at least 24 h. The specimens were processed in an automated tissue processor (Leica TP 1020, Germany), embedded in paraffin wax, cut into thin sections (4 µm) using a rotary microtome (Leica Jung Multicut 2045, Germany), and stained with Harris´s hematoxylin and eosin (H&E) stain. The stained sections were mounted with DPX and observed using a light microscope (Nikon Eclipse 50i, Japan) to assess the histopathological changes including congestion, hemorrhage, infiltration of the inflammatory cells, and necrosis.

The pathological inflammation scoring (PIS) system in tissues throughout acute inflammation was projected as the sum of scores from every group for that individual where S0 = normal, S1 = mild, S2 = moderate, S3 = severe [20].

**Ethical consideration:** animal experiments were approved by the Institutional Animal Care and Use Committee (IACUC) based on the Universiti Putra Malaysia Code of Practice for Care and Use of Animals for Scientific Purposes (UPM/IACUC/AUP-R047).

**Statistical analysis:** the data analysis was conducted using SPSS statistical analysis software (SPSS V-23). The serum biomarkers were expressed as mean ± S.D using one-way ANOVA and a posthoc test for multiple comparisons based on the normally distributed sets of data was conducted. The mean values were compared between groups by non-parametric analysis of variance using the Kruskal-Walli´s test, followed by standard process comparisons. Differences between groups were measured to be statistically significant when p < 0.05.

## Results

**Efficacy of peptide treatment against *P. aeruginosa* sepsis:** the mortality rate of mice in groups after infection with LD100 of MDR *P. aeruginosa* revealed a significant difference (p<0.05) in the mortality rate between the PBS control treatment group G2 (100%) and the treated groups G3 (25%), G4 (50%), and G5 (12.5%) (Table 1). Similarly, the MDR *P. aeruginosa* burden in the internal organs and blood samples of PBS control and treatment groups shows that the PBS control group (G2) had a significantly (p < 0.05) higher number of bacteria with the lung being the infected organ. However, the bacterial burden in the lung, liver, and spleen although was significantly lower in the entire treated groups (G3, G4, and G5), and no statistically significant difference was observed between the two treatment groups (G3 and G5). However, there was a significant (p < 0.05) difference in bacterial count between G4 and other treated groups (G3 and G5) in the liver and spleen. On the other hand, the blood cultures in the three treated groups of G3, G4, and G5 showed a decrease in the bacterial count when compared to the PBS control group G2 (Table 1).

**Table 1:** bacterial counts in the internal organs (lung, liver, spleen) and blood from mice for a group of phosphate buffered saline (PBS) control and groups treated with antimicrobial peptides (AMP) and ciprofloxacin (CE)

G*	L%**	Mean ± S.D bacterial count (CFU/g)			Mean ± S.D bacterial count (CFU/mL)
		Lung	Liver	Spleen	Blood
G2	100B	3.16x10^8C^ ± 1.57x10^7^	2.8 x10^7C^ ± 2x 10^6^	3.8x10^7C^ ± 6.8x10^6^	3.3x10^5B^ ± 3.05x10^4^
G3	25A	4.95x10^5B^ ± 7.3x10^4^	7.5 x10^5A^ ± 1.2x10^5^	2.4x10^6A^ ± 3.5x10^5^	7.46x10^2A^ ± 61.1
G4	12.5A	3.4x10^6A^ ± 5.2x10^6^	2.76x10^6B^ ±1.1x10^5^	1.9x10^6B^ ± 1.5x10^6^	7.4x10^3A^ ± 1.5x10^2^
G5	50A	7.38x10^4A^ ± 3.1x10^6^	4.5 x10^5A^ ± 3 x 10^4^	1.7x10^5A^ ± 6.8x10^5^	5.9 x10^2A^ ± 5.4x10^2^

Different letters (A, B, C) indicate a significant difference (p < 0.05) among treatment groups: statistical comparisons between groups were corrected according to Bonferroni's criterion; G: group, each value represents the mean value from three determinations ± standard derivation (SD)

Bacterial burden in lung, liver, and spleen showed a significant decrease (p < 0.05) in the entire treated groups of G3, G4, and G5 when compared with the non-treated group G2. There was no significant difference between the two treated groups of G3 and G5; otherwise, there was a significant (p < 0.05) difference in bacterial count between G4 and other treated groups (G3 and G5) in the liver and spleen. The bacterial count for the lungs in G3, G4, and G5 was significantly different (p < 0.05) from G2. There was no significant difference between G4 and G5. Nonetheless, there was a significant (p < 0.05) difference between G3 and other treated groups (G4 and G5). The blood cultures in the three treated groups of G3, G4, and G5 revealed a significant decrease in the bacterial count when compared to the G2 and this difference was not statistically significant (p >0.05).

**Figure 2:**
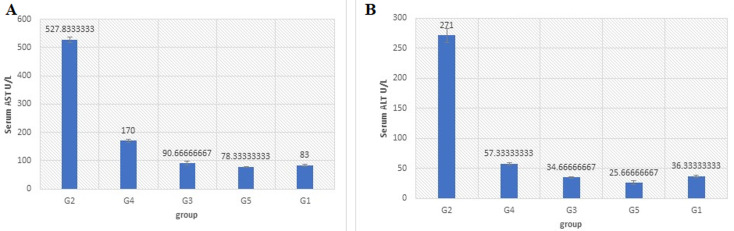
A,B) the mean serum AST and ALT concentration of Institute of Cancer Research (ICR) mice after 24h of last treatment; a significant difference (p < 0.05) between control and other groups were indicated by different letters (error bar = S.D)

**Serum biochemistry:** the level of serum transaminase enzymes (AST U/L and U/LALT) in G2 (528 ± 16.2; 227 ± 21) was significantly higher when compared with normal group G1 (80 ± 5; 36.3 ± 3.786). However, in G3, G4, and G5 (113.3 ± 0.7; 116.3 ± 23.4; 117.3 ± 21.57, respectively) the concentration was significantly (p< 0.05) higher than G1, though the difference between the three groups (G3, G4, and G5) was not statistically significant (Figure 2A). The levels of AST in G2, G3, G4, and G5 were 6.6,1.4, 1.45, 1.46-fold, respectively, higher than G1. ALT levels (Figure 2B) in G3, G4, and G5 (62.3 ± 8.08; 65±19.5; 40 ± 4.6, respectively) were significantly (p < 0.05) different from G2. Otherwise, there was no significant difference between the three groups G3, G4, and G5. Levels of ALT in G2, G3, G4, and G5 were 7.6, 1.7, 1.79, and 1.1-fold, respectively, higher than G1.

**Figure 3:**
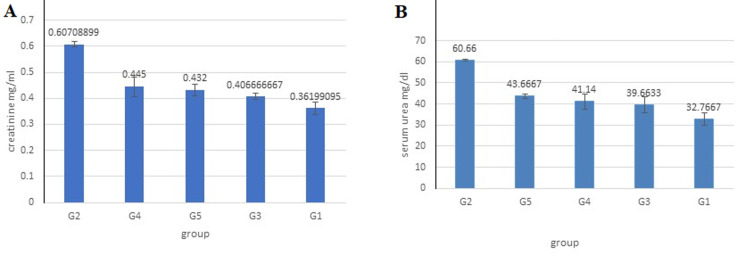
A,B) the mean of serum creatinine and urea concentration of ICR mice after 24h of last treatment; a significant difference (p < 0.05) between control and other groups were indicated by different letters (error bar = S.D)

Evaluation of the creatinine levels mg/dl (Figure 3A) in G3 (0.61 ± 0.017) revealed a significant (p <0.05) increase as compared to the control G1 (0.362 ± 0.04) and treated groups G3, G4, and G5 (0.406 ± 0.03; 0.43 ± 0.037; 0.44 ± 0.065, respectively). However, G3, G4, and G5 revealed no significant difference from G1. Levels of creatinine in groups G2, G3, G4, and G5 were 1.6, 1.1, 1.18, and 1.2-fold, respectively, higher than G1. In the case of urea levels (Figure 3B), G2 (60.6 ± 0.57) revealed a significant (p < 0.05) difference from G1 (32.76 ± 5.26). However, in G3, G4, and G5 (43.6 ± 1.5; 41.14 ± 6.3; 39.6 ± 6.8), there was no significant difference between them and when compared to G1. Otherwise, G3, G4, and G5 revealed a significant (p < 0.05) decrease in urea level compared to G2. The levels of urea in groups G2, G3, G4, and G5 were 1.8, 1.3, 1.2, and 1.2-fold, respectively, higher than G1.

**Figure 4:**
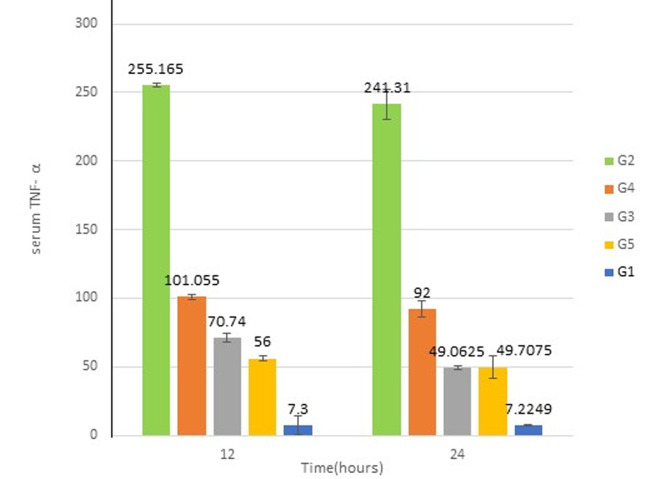
serum levels of TNF-α at 12 and 24 h during the treatment period; different letters indicate significant differences (p < 0.05) between groups (error bar = S.D)

**Figure 5:**
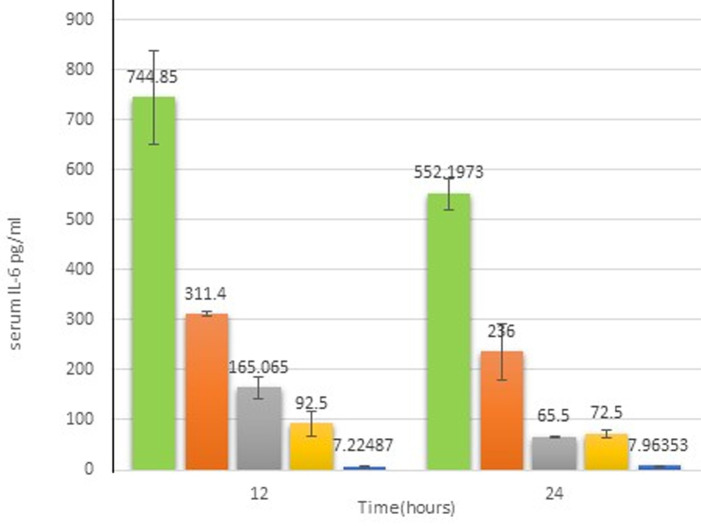
serum levels of IL-6 at 12 and 24 h during the treatment period; different letters indicate significant differences (p < 0.05) between groups (error bar = S.D)

**Pro-inflammatory cytokines (TNF-α and IL-6):** Figure 4 shows that the levels of TNF-α pg/mL after 12 h in G2 mice (255.16 ± 1.58) was significantly (p < 0.05) higher than G1 (7.0 ± 1). Whereas, TNF-α levels in G3, G4, and G5 (70.74 ± 3.16; 56 ± 1.2; 101.05 ± 1.6, respectively) were significantly (p < 0.05) different from the PBS-control G2 group and this difference was found to be significant (p < 0.05). Similarly, the TNF-α after 24 h, in G2 (241.31 ± 11.23) was significantly (p < 0.05) higher than G1 (6.9 ± 0.05). Whereas, TNF-α levels in G3, G4, and G5 (49.06 ± 1.56; 92 ± 6; 49.7 ± 7.9) were significantly (p < 0.05) decreased compared to G2. The levels of TNF-α after 24 h in G2, G3, G4, and G5 were 34.4,7, 9, and 7.1-fold, respectively, more than the level of TNF-α in the control group. Furthermore, Figure 5 illustrates the levels of IL-6 pg/mL after 12 h. In G2 (744.85 ±131.734) was significantly (p < 0.05) higher compared to G1 (7.23 ± 0.99), whereas IL-6 levels in treated groups G3, G4, and G5 (92.5 ± 34.64; 311 ±7.63; 165.06, respectively) were significantly (p < 0.05) different from G2. Likewise, G4 showed a significant difference (p < 0.05) from G3 and G5. The entire treated groups were significantly (p < 0.05) different from G1. As well, the levels of IL-6 after 24 h show levels of IL-6 in G2 (552.197 ± 44.11) were significantly (p < 0.05) higher compared to G1 (7.9 ± 0.048), while IL-6 levels in treated groups G3 and G5 (72.5 ± 12.02; 65.5 ± 2.12) were significantly (p< 0.05) different from G2. Likewise, G4 (236 ± 80.6) was significantly different (p < 0.05) from treated groups (G3 and G5) and control groups (G1 and G2). The levels of IL-6 after 24 h in G2, G3, G4, and G5 were 69-, 9-, 7-, and 29.5-fold, respectively, higher than the level of IL-6 in the control group.

**Figure 6:**
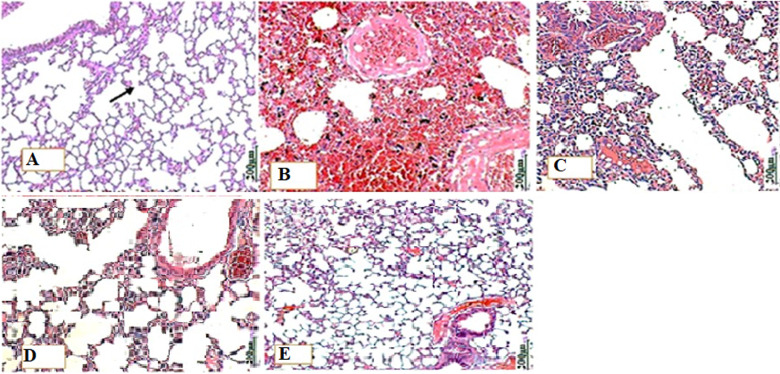
microphotographs of ICR mice lungs with black arrow indicating alveolar ducts: A) showing normal histological properties; B) showing thickening of the alveolar wall and pulmonary hemorrhage; C,D,E) display a gradual decrease in the degree of congestion and hemorrhage (H&E X20 magnification)

**Histopathology examination:** the histological examination showed the normal lung of the G1 group (Figure 6A), showing well-defined respiratory bronchioles (RB) with numerous alveolar (arrow) and alveolar sac (S). but there were histological changes in the lungs of treated groups G3, G4, and G5 (Figure 6(B,C,D,E)) in the level of vascular congestion and hemorrhage with a slight thickening of the alveolar and a gradual decrease in the degree of congestion, unlike the lung of untreated mice. PIS scores for all treatment groups were moderate.

**Figure 7:**
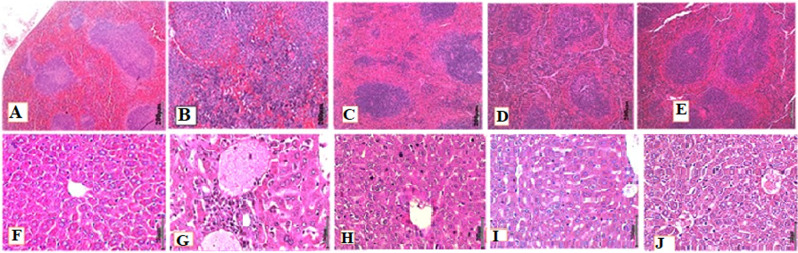
microphotographs of ICR mice spleens and liver: A) showing normal histological architecture; B) showing loss of the typical structures of the germinal centers with congested blood vessels; C,D,E) display the red and white pulps and germinal centers with moderate histological changes (H&E X20 magnification); F) showing normal histological properties with black arrow indicating the sinusoid; showing a severe massive infiltration of inflammatory cells, severe hemorrhage, and hepatocellular necrosis, with black arrow pointing at the sinusoid (S); H,I,J) showing a slightly reduced inflammation and congestion (H&E X20 magnification)

The spleen from the control group G1 (Figure 7A) shows normal histological architectures with normal red and white pulps. In the PBS control group G2 (Figure 7B), mice spleen displayed severe inflammation; the red and white pulps were enlarged and accompanied with the loss of the typical structures of the germinal centers, with congested blood vessels, hemorrhage, and infiltration of inflammatory cells, showing many erythrocytes in the red pulp. There were also histological changes in the spleens of treated groups G3, G5, and G4 (Figure 7(C,D,E)), showing congested blood vessels, hemorrhage, and infiltration of inflammatory cells. Unlike the spleen of untreated mice, treated groups showed a gradual decrease in the degree of congestion and damage. PIS scores for G3 and G5 were mild and were moderate for G4.

Finally, the liver of G1 mice (Figure 7F) showed normal histological architectures of hepatocytes, where normal hepatocytes (arrow) were arranged in plates around the central vein (CV) and sinusoids (S) between plates. Liver tissue from G2 mice (Figure 7G) showed a severe massive infiltration of inflammatory cells like neutrophils and macrophages in the portal area, obstruction of sinusoids, congested blood vessels, severe hemorrhage, and hepatocellular necrosis. Similarly, there were histological changes in the liver of mice from treated groups G3, G5, and G4 (Figure 7(H,I,J)), showing congested blood vessels, infiltration of inflammatory cells, and hemorrhage. The histology of liver from groups G3 and G5 mice showed nonspecific lesions with significantly reduced hepatic degeneration with a marked reduction of intact hepatic lobules in the hemorrhage, inflammation, and marked hepatic regeneration (binucleate liver cells). There was a slight reduction of inflammation and congestion. Inflammation scoring for G3 and G5 were mild and was moderate for G4.

## Discussion

In this study, the antimicrobial peptides obtained from climbing perch were evaluated for their antibacterial activities against multi-drug resistant aquatic pathogenic bacteria (*P. aeruginosa*), and it was observed to exhibit potent antibacterial activities against the bacteria and compared favorably with ciprofloxacin in terms of treatment outcome.

Compound efficacy of the AMP was assessed by survival rate, bacterial cultures, and cytokine levels as described by Cirioni *et al*. [21], while the death rate was utilized as the gold standard to evaluate therapeutic properties [21]. Additionally, the principal determinants of sepsis are bacterial clearance and inflammatory reaction to the disease [21]. The clearance of bacteria is the most significant criterion for the survival of patients with sepsis [22,23]. In our investigation, the rate of mortality for the PBS-control group G2 was 100%, indicating severe sepsis after systemic infection with multidrug-resistant (MDR) *P. aeruginosa*. An extremely virulent strain that is resistant to phagocytosis and grows in the peritoneal cavity of the mouse enters the blood system and invades the organs, which eventually leads to the death of the mice as a result of septicemia [1]. The mortality in the AMP-treated group significantly decreased to 25% and 12.5%, respectively. This implies that the interventions were successful in halting the lethal consequence of lipopolysaccharide (LPS) and other virulence factors of MDR *P. aeruginosa*. This finding aligned with the previous reports where oral administration of Tilapia Piscidin 4 (TP4) against multidrug-resistant gastric pathogen *H. pylori* was found to be protective [24]. On the other hand, the mortality rate of mice in group G4 treated with ciprofloxacin was 50%. This shows that the antibiotic has a direct effect on bacteria but may not have prevented the LPS effect, especially because bacteria are known to utilize diverse strategies in resisting antibiotics.

Concerning the burden of infection, the treatment using AMP leads to a significant decrease of MDR *P. aeruginosa* burden in organs (liver, lung, and spleen) and blood for the two treated groups G3 and G5 compared to the non-treated group G2. Our previous finding recorded that bioactive crude and ciprofloxacin were active against MDR *P. aeruginosa* in-vitro; however, there is variety between in-vitro and in-vivo linked to the minimum inhibitory concentrations calculated for killing in-vitro assays are very hard to attain in-vivo [25]. Consequently, antimicrobial proteins and peptides most likely act not exclusively by direct killing of the pathogen among the establishment of immune protective circuits [25]. G3 group was treated after 10 min of infection in the same way as A3-APO and epinecidin-1, these compounds demonstrated a defensive effect when they were administered to mice right away after infection with MDR *P. aeruginosa* [26]. However, presently, we do not know if cationic peptides and proteins in the crude are cleaved in the gut or the blood and predicate antibacterial peptides which were analysed by liquid chromatography-mass spectrometry (LC-MS) can generate during digestion in the gut of mice. Previous reports have shown that bioactive peptides were resistant to the action of peptidases [27,28]. Based on the duration of treatment, the AMP did not lose the action and it was stable after oral administration as shown by the number of bacteria decreasing significantly when compared with the non-treatment group G2 and antibiotic group G4. It is important to note that, despite there are many AMPs that retain their activity profile after long exposure, experimental data indicate the possibility of developing resistance against AMPs due to sustained selection pressure [29,30].

This investigation also noticed an increase of serum AST and ALT in the non-treated group which conforms with earlier studies. In the liver, several local and systemic defense mechanisms of the innate immune system are initiated [31]. Therefore, the hepatic response in sepsis is contributed by four major cell types including Kupffer cells, neutrophils, hepatocytes, and liver sinusoidal endothelial cells [32]. Endotoxin is a constituent of the outer membrane of gram-negative bacteria which is implicated in the pathogenesis of sepsis; Kupffer cells are significantly involved in these processes, including the phagocytosis of bacteria, secretion of pro-inflammatory and anti-inflammatory cytokines, and the employment of other immune cells to the liver, for example, neutrophils or monocytes [31]. These findings are related to significant rates of hepatocellular apoptosis and increased plasma levels of alanine aminotransferase (ALT) and aspartate aminotransferase (AST) that are present in severe hepatic injury [31,33]. The ALT and AST levels in the non-treated group were extremely high, indicating systematic infection of MDR *P. aeruginosa*, which results in liver malfunction. Organ malfunctions are characterized by a high number of biochemical markers of tissue injury [34]. Furthermore, the typical indicators of progressing infections are liver enzymes and renal function parameters [35,36]. In this study, the level of ALT and AST showed a significant reduction in the G4 group (CE-treated group), compared with the non-treated group G2. These results conform with Yagel *et al*. (1996) who reported that oral administration of ciprofloxacin actively kills *P. aeruginosa* in-vivo. Moreover, the levels of ALT and AST in both AMP-treated groups (G3 and G5) were significantly low, and this may imply that the AMPs by their antimicrobial proteins and peptides prevented the damaging effect of MDR *P. aeruginosa* of the liver cells. This finding is in agreement with the results recorded by Lee *et al*. [37] which employed epinecidin-1, fish AMPs isolated from *Epinephelus coioides*, for treating mice infected with *P. aeruginosa*. They revealed that epinecidin-1 might be involved in defending the liver against LPS-induced dysfunction in preventing injury to numerous other organs in septic mice and attenuated the increases in plasma ALT and AST after *P. aeruginosa* infection. In addition, serum creatinine and urea levels were not high in the non-treated group. As reported by Bhargava *et al*. [38], loss of kidney function could take place in sepsis without much alteration in the urea or creatinine level., serum levels of urea and creatinine in G3, G4, and G5 seemed to be normal and were the same as the level in control group G1.

Lipopolysaccharide (LPS) among many other components of gram-negative bacteria was binding to Toll-like receptor 4 [39]. These binding initiates host immune responses such as the expression of pro-inflammatory cytokines, TNF-α and IL-6 [40]. In this study, the concentrations of TNF-α and IL-6 were measured to better understand the immune response in our mouse models. Different massive inflammation in the non-treated group (G2) was detected through TNF-α and IL-6 levels, which considerably increased (33- and 85-fold) compared to control group G1. TNF-α is considered to be the master regulator of pro-inflammatory cytokine production and regulates several facets of macrophage function [40]. IL-6 on the other hand is produced by a broad range of cells, such as macrophages, as a result of stimulation with LPS, IL-1, and TNF-α in immune responses [40]. These cytokines are fundamentally accountable for the features of systemic inflammatory response syndrome (SIRS) and can potentially be of use as biomarkers of sepsis [40]. TNF-α and IL-6 levels in the AMP-treated groups (G3 and G5) were significantly reduced after 12 and 24 h. After 24 h, the levels of TNF-α and IL-6 in groups G3 and G5 were 7 and 7.1 and 9 and 7 folds respectively, higher than the control group. This is a finding similar to previous reports by Hancock and Scott (2000) that cationic peptides act on gram-negative bacteria by initially binding to their surface polyanionic LPS followed by self-promoted uptake across the outer membrane [41,42]. Coincidentally, such peptides inhibit the production of cytokines such as TNF-α and IL-6 by macrophages stimulated with LPS. Generally, antimicrobial peptides possess the capability to decrease pro-inflammatory reactions through direct interaction with bacterial membrane and pathogen-associated molecular patterns (PAMPs), making them unrecognizable to pattern recognition receptors, hence preventing phagocyte activation and secretion of pro-inflammatory cytokines [43]. Mice in group G4 were treated with the conventional antibiotic ciprofloxacin, which is commonly utilized to protect mice from lethality against infections caused by *P. aeruginosa*. Ciprofloxacin and levofloxacin are the only commercially available fluoroquinolones that are approved by the Food and Drug Administration for the treatment of systemic *P. aeruginosa* infections [44]. The finding here of TNF-α and IL-6 levels in group G4 were 9 and 29.57 respectively fold higher than recorded in the normal group. In general, antibiotic ciprofloxacin after killing the gram-negative bacteria could encourage the discharge of LPS, which binds to LPS-binding protein (LBP) in the blood and transfers it to the CD14 receptor on the surface of immune cells. The complex then starts intracellular signal reactions that induce the production of inflammatory cytokines [43].

Finally, the pathological changes in tissues and organs reveal the rigorousness of sepsis by congestion, inflammatory cells, abscess, or necrosis. Inflammation is characterized by histological alterations in the lungs, liver, and other organs. Infection, which leads to extreme pro-inflammatory reactions, could result in a systemic inflammatory reaction, thereby promoting injury of the organ [36]. Inflammatory cell infiltrations were recorded in the lungs of mice from group G2 because of *P. aeruginosa* infection and possible function as phospholipases A2 (PLA2). PLA2 are lipolytic enzymes that hydrolyze membrane phospholipids and release fatty acids, mainly arachidonic acid (AA) that plays a vital role in host cell deterioration and necrosis [44]. In the same vein, the spleen is also known to be a vital organ in clearing senescent erythrocytes and maintenance of blood reserve and also plays a major role in the immune system [45]. A large number of erythrocytes in the red pulp of the G2 spleen may be indicative of septic spleens [46]. The enormous splenic erythrocyte accretion in G2 was comparable to the previous study on LPS-treated mice; the accumulation likely contributed to spleen swelling. LPS challenge led to a redeployment of erythrocytes into the spleen, which could show damage to the red blood cells [46]. The extreme inflammatory reaction leads to hepatic cell necrosis and apoptosis, which leads to liver damage.

Furthermore, histopathological analysis was also conducted on the liver due to its role as a lymphoid organ in reaction to sepsis, acting as a double-edged weapon in sepsis; liver-mediated immune response is accountable for clearance of bacteria and toxins; however, it also causes inflammation, immunosuppression, and organ damage [36]. The liver of mice from group G2 showed severe infiltration of inflammatory cells, for example, neutrophils and macrophages, in the portal area, establishing the severe inflammation by *P. aeruginosa*. The distinctive morphological features of bacterial infection of persisting inflammation are moderate lymphocytic and neutrophilic portal infiltration, hepatocyte injury, and necrosis. The bacterial burden in organs decreased in the three treated groups G3, G4, and G5, which proved the ability of this agent to reduce bacterial growth. The number of bacteria was significantly reduced for AMP groups G3 and G5 compared to ciprofloxacin group G4. As well, a significant decrease in serum IL-6 and TNF-α levels confirmed of neutralization of LPS from MDR *P. aeruginosa*. Therefore, the magnitude of histological changes was alleviated from severe to mild in the spleen and liver. Meanwhile, the histological changes in the lungs were moderate, which showed high numbers of bacteria.

**Limitation:** this investigation was not able to evaluate the efficacy of the AMPs in relation to the commonly used antimicrobials in aquaculture. Secondly, full characterization of the peptides would have shed light into the mechanism by which these peptides are acting against the pathogenic bacteria.

## Conclusion

The data presented in this investigation indicate that AMP significantly (p < 0.05) reduced the mortality rate and stopped the lethal effect of multidrug-resistant (MDR) *P. aeruginosa* in ICR mice compared to the PBS-control group, and the group that received treatment with ciprofloxacin. Furthermore, AMP reduced the bacterial burden in the internal organs and blood samples within 48 h. AMPPC also significantly (p < 0.05) reduced the levels of pro-inflammatory cytokines (TNF-α and IL-6), liver enzymes (AST and ALT) and renal function biomarkers (creatinine and urea). On the other hand, clearance of bacteria and neutralization of LPS by AMPPC reduced acute inflammation of internal organs (liver, spleen, and kidney) from severe to mild. In vivo, the AMPs markedly alleviated multidrug-resistant *P. aeruginosa* with an enhanced therapeutic.

### What is known about this topic

- *Piscidins, and cathelicidins are linear AMPs that have been found to disrupt pathogenic bacterial membranes;*
- *These properties of AMPs effectively reduce the possibility of development of resistance against bacteria*.

### What this study adds

- *AMP significantly reduced the lethal effect of MDR P. aeruginosa;*
- *The fish AMPs showed superior antimicrobial activities compared to ciprofloxacin against aquatic pathogenic multidrug-resistant bacteria*.

## References

[1] Cigana C, Ranucci S, Rossi A, De Fino I, Melessike M, Bragonzi A (2020). Antibiotic efficacy varies based on the infection model and treatment regimen for Pseudomonas aeruginosa. Eur Respir J.

[2] Gyawali R, Ibrahim SA (2014). Natural products as antimicrobial agents. Food Control.

[3] Hayashi MA, Bizerra FC, Da Silva PI (2013). Antimicrobial compounds from natural sources. Front Microbiol.

[4] Citarasu T (2012). Natural antimicrobial compounds for use in aquaculture. Infectious Disease in Aquaculture.

[5] Al-Rasheed A, Handool KO, Alhelli AM, Garba B, Muhialdin BJ, Masomian M (2020). Assessment of Some Immune Components from The Bioactive Crude Extract Derived from The Epidermal Mucus of Climbing Perch Anabas Testudines. Turkish Journal of Fisheries and Aquatic Sciences.

[6] Al-Rasheed A, Handool KO, Garba B, Noordin MM, Bejo SK, Kamal FM (2018). Crude extracts of epidermal mucus and epidermis of climbing perch Anabas testudineus and its antibacterial and hemolytic activities. The Egyptian Journal of Aquatic Research.

[7] Rakers S, Niklasson L, Steinhagen D, Kruse C, Schauber J, Sundell K (2013). Antimicrobial peptides (AMPs) from fish epidermis: perspectives for investigative dermatology. J Invest Dermatol.

[8] Aden MA, Bashiru G (2022). HOW MISUSE OF ANTIMICROBIAL AGENTS IS EXACERBATING THE CHALLENGES FACING SOMALIA´S PUBLIC HEALTH. Afr J Infect Dis.

[9] Algammal AM, Mohamed MF, Tawfiek BA, Hozzein WN, El Kazzaz WM, Mabrok M (2020). Molecular Typing, Antibiogram and PCR-RFLP Based Detection of Aeromonas hydrophila Complex Isolated from Oreochromis niloticus. Pathogens.

[10] Moussa AA, Abdulahi Abdi A, Awale MA, Garba B (2021). Occurrence and Phenotypic Characterization of Multidrug-Resistant Bacterial Pathogens Isolated from Patients in a Public Hospital in Mogadishu, Somalia. Infect Drug Resist.

[11] Behzadi P, Baráth Z, Gajdács M (2021). It´s Not Easy Being Green: A Narrative Review on the Microbiology, Virulence and Therapeutic Prospects of Multidrug-Resistant Pseudomonas aeruginosa. Antibiotics (Basel).

[12] Gajdács M (2019). The Concept of an Ideal Antibiotic: Implications for Drug Design. Molecules.

[13] Garba B, Habibullah SA, Saidu B, Suleiman N (2019). Effect of mastitis on some hematological and biochemical parameters of Red Sokoto goats. Vet World.

[14] Hazis NHA, Ahmad N, Zakaria Z, Hassan L, Sharif Z, Ali RM (2022). Pulsed-Field Gel Electrophoresis Analysis of Salmonella enterica Serovar Enteritidis from Human and Food in Malaysia. International Journal of Infectious Diseases.

[15] Zakaria Z, Hassan L, Sharif Z, Ahmad N, Mohd Ali R, Amir Husin S (2022). Virulence Gene Profile, Antimicrobial Resistance and Multilocus Sequence Typing of Salmonella enterica Subsp. enterica Serovar Enteritidis from Chickens and Chicken Products. Animals (Basel).

[16] Chen Y, Wu J, Cheng H, Dai Y, Wang Y, Yang H (2020). Anti-infective Effects of a Fish-Derived Antimicrobial Peptide Against Drug-Resistant Bacteria and Its Synergistic Effects With Antibiotic. Front Microbiol.

[17] Reed LJ, Muench H (1938). A simple method of estimating fifty per cent endpoints. American Journal of Epidemiology.

[18] Conlon JM (2007). Purification of naturally occurring peptides by reversed-phase HPLC. Nat Protoc.

[19] Lamont IL, Martin LW (2003). Identification and characterization of novel pyoverdine synthesis genes in Pseudomonas aeruginosa. Microbiology.

[20] Wu D, Zhou S, Hu S, Liu B (2017). Inflammatory responses and histopathological changes in a mouse model of Staphylococcus aureus-induced bloodstream infections. J Infect Dev Ctries.

[21] Cirioni O, Silvestri C, Ghiselli R, Orlando F, Riva A, Gabrielli E (2009). Therapeutic efficacy of buforin II and rifampin in a rat model of Acinetobacter baumannii sepsis. Crit Care Med.

[22] Nesseler N, Launey Y, Aninat C, White J, Corlu A, Pieper K (2016). Liver Dysfunction Is Associated with Long-Term Mortality in Septic Shock. Am J Respir Crit Care Med.

[23] Strnad P, Tacke F, Koch A, Trautwein C (2017). Liver-guardian, modifier and target of sepsis. Nat Rev Gastroenterol Hepatol.

[24] Narayana JL, Huang H-N, Wu C-J, Chen J-Y (2015). Efficacy of the antimicrobial peptide TP4 against Helicobacter pylori infection: in vitro membrane perturbation via micellization and in vivo suppression of host immune responses in a mouse model. Oncotarget.

[25] Silva T, Gomes M (2017). Immuno-Stimulatory Peptides as a Potential Adjunct Therapy against Intra-Macrophagic Pathogens. Molecules.

[26] Pan C-Y, Chen J-C, Sheen J-F, Lin T-L, Chen J-Y (2014). Epinecidin-1 Has Immunomodulatory Effects, Facilitating Its Therapeutic Use in a Mouse Model of Pseudomonas aeruginosa Sepsis. Antimicrob Agents Chemother.

[27] Daliri E, Oh D, Lee B (2017). Bioactive Peptides. Foods.

[28] Rafiq S, Gulzar N, Sameen A, Huma N, Hayat I, Ijaz R (2021). Functional role of bioactive peptides with special reference to cheeses. Int J Dairy Technol.

[29] Lofton H, Pränting M, Thulin E, Andersson DI (2013). Mechanisms and fitness costs of resistance to antimicrobial peptides LL-37, CNY100HL and wheat germ histones. PLoS One.

[30] Spohn R, Daruka L, Lázár V, Martins A, Vidovics F, Grézal G (2019). Integrated evolutionary analysis reveals antimicrobial peptides with limited resistance. Nat Commun.

[31] Traeger T, Mikulcak M, Eipel C, Abshagen K, Diedrich S, Heidecke C-D (2010). Kupffer cell depletion reduces hepatic inflammation and apoptosis but decreases survival in abdominal sepsis. Eur J Gastroenterol Hepatol.

[32] Wang D, Yin Y, Yao Y (2014). Advances in sepsis-associated liver dysfunction. Burns Trauma.

[33] Rangel JM, Sparling PH, Crowe C, Griffin PM, Swerdlow DL (2005). Epidemiology of Escherichia coli O157: H7 Outbreaks, United States, 1982-2002. Emerg Infect Dis.

[34] Duran-Bedolla J, Montes de Oca-Sandoval MA, Saldaña-Navor V, Villalobos-Silva JA, Rodriguez MC, Rivas-Arancibia S (2014). Sepsis, mitochondrial failure and multiple organ dysfunction. Clin Invest Med.

[35] Lala V, Zubair M, Minter DA (2023). Liver Function Tests 2023 Jul 30. StatPearls [Internet].

[36] Minemura M, Tajiri K, Shimizu Y (2014). Liver involvement in systemic infection. World J Hepatol.

[37] Lee MT, Sun TL, Hung WC, Huang HW (2013). Process of inducing pores in membranes by melittin. Proc Natl Acad Sci U S A.

[38] Bhargava R, Altmann CJ, Andres-Hernando A, Webb RG, Okamura K, Yang Y (2013). Acute lung injury and acute kidney injury are established by four hours in experimental sepsis and are improved with pre, but not post, sepsis administration of TNF-α antibodies. PLoS One.

[39] Vaure C, Liu Y (2014). A comparative review of toll-like receptor 4 expression and functionality in different animal species. Front Immunol.

[40] Kany S, Vollrath JT, Relja B (2019). Cytokines in Inflammatory Disease. Int J Mol Sci.

[41] Hancock RE, Sahl HG (2006). Antimicrobial and host-defense peptides as new anti-infective therapeutic strategies. Nat Biotechnol.

[42] Hancock RE, Scott MG (2000). The role of antimicrobial peptides in animal defenses. Proc Natl Acad Sci U S A.

[43] Rosenfeld Y, Shai Y (2006). Lipopolysaccharide (Endotoxin)-host defense antibacterial peptides interactions: role in bacterial resistance and prevention of sepsis. Biochim Biophys Acta.

[44] Kirschnek S, Gulbins E (2006). Phospholipase A2 functions in Pseudomonas aeruginosa-induced apoptosis. Infect Immun.

[45] Ashare A, Monick MM, Powers LS, Yarovinsky T, Hunninghake GW (2006). Severe bacteremia results in a loss of hepatic bacterial clearance. Am J Respir Crit Care Med.

[46] Seemann S, Zohles F, Lupp A (2017). Comprehensive comparison of three different animal models for systemic inflammation. J Biomed Sci.

